# Oceanalin B, a Hybrid α,ω-Bifunctionalized Sphingoid Tetrahydroisoquinoline β-Glycoside from the Marine Sponge *Oceanapia* sp.

**DOI:** 10.3390/md19110635

**Published:** 2021-11-12

**Authors:** Tatyana N. Makarieva, Natalia V. Ivanchina, Pavel S. Dmitrenok, Alla G. Guzii, Valentin A. Stonik, Doralyn S. Dalisay, Tadeusz F. Molinski

**Affiliations:** 1 G.B. Elyakov Pacific Institute of Bioorganic Chemistry, Far Eastern Branch of the Russian Academy of Sciences, Pr. 100-Let Vladivostoku 159, 690022 Vladivostok, Russia; ivanchina@piboc.dvo.ru (N.V.I.); paveldmt@piboc.dvo.ru (P.S.D.); gagry@rambler.ru (A.G.G.); 2 Department of Chemistry and Biochemistry/SSPPS, University of California, San Diego, CA 92093-0358, USA; ddalisay@usa.edu.ph (D.S.D.); tmolinski@ucsd.edu (T.F.M.); 3 Center for Chemical Biology and Biotechnology (C2B2), Department of Biology, College of Liberal Arts, Sciences and Education, University of San Agustin, Iloilo City 5000, Philippines

**Keywords:** *Oceanapia* sp., marine sponge, oceanalin B, bipolar sphingolipids, tetrahydroisoquinoline, glycoside, antifungal activity

## Abstract

Oceanalin B (**1**), an α,ω-bipolar natural product belonging to a rare family of sphingoid tetrahydoisoquinoline β-glycosides, was isolated from the EtOH extract of the lyophilized marine sponge *Oceanapia* sp. as the second member of the series after oceanalin A (**2**) from the same animal. The compounds are of particular interest due to their biogenetically unexpected structures as well as their biological activities. The structure and absolute stereochemistry of **1** as a α,ω-bifunctionalized sphingoid tetrahydroisoquinoline β-glycoside was elucidated using NMR, CD and MS spectral analysis and chemical degradation. Oceanalin B exhibited in vitro antifungal activity against *Candida* *glabrata* with a MIC of 25 μg/mL.

## 1. Introduction

A family of highly modified α,ω-bipolar sphingolipid–like natural products has been previously described from the marine sponges *Rhizochalina incrustata* [1,2,3], *Oceanapia* sp. [4], *Oceanapia phillipensis* [5], *Calyx* sp. [6], *Leucetta microraphis* [7], *Cladocroce* sp. [8] and an unidentified Australian sponge [9]. These compounds are of particular interest due to biogenetically unexpected structures. All of them are aminolipids, consisting of symmetrical or almost-symmetrical long hydrocarbon chains (C28–C30), functionalized at both ends as vicinal amino alcohols. Some of these substances are glycosylated with a glucose or galactose residue [1,2,3,4,5,6,8]. α,ω-Bipolar sphingolipids substances exhibit antifungal [4,5,10,11], antimicrobial [1] and cytotoxic activity against mouse Ehrlich carcinoma cells [1]; DNA-damaging activity [6] and inhibition of protein kinase C activity [9]. In continuation of our search for new antifungal agents against the pathogenic fluconazole-resistant yeast *Candida glabrata*, we isolated oceanalin A (**2**) [4], a unique α,ω-bipolar compound containing isoquinoline and sphingolipid units on the ends of the molecule. The remarkable finding from our analysis of the structure of oceanalin A is an unprecedented confluence of sphingolipid and isoquinoline pathways in natural product biosynthesis [4]. Herein, we report the isolation and structure elucidation of another related alkaloidal lipid **1,** which we designated as oceanalin B. Oceanalin B contains an acid-labile allylic hydroxyl group and may be considered as a genuine natural product, while oceanalin A is likely a solvolysis artefact formed during extraction and isolation procedures.

## 2. Results and Discussion

**Figure 1:**
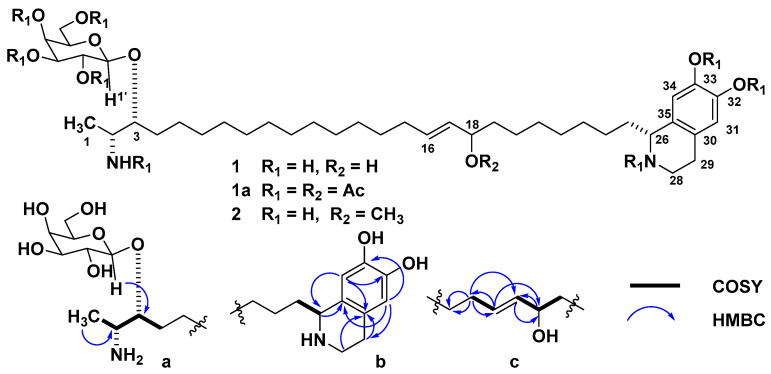
The structures of oceanalins B and A (**1**, **2**) and oceanalin B peracetate **1a**. (**a**–**c**) Key COSY and HMBC correlations of fragments of oceanalin B.

The EtOH extract of the lyophilized sponge was concentrated and partitioned between aqueous EtOH and hexane. The aqueous EtOH layer was further partitioned against *n*-BuOH, and the *n*-BuOH-soluble materials were separated by Polychrom-1 flash chromatography and reversed-phase HPLC (YMC-Pack ODS-A column, MeOH–H_2_O–TFA, 80:20:0.1%) to provide oceanalin B (**1**) as a pale yellow glass (yield 0.03% based on dry weight of the sponge), along with known rhizochalin (**3**). Compound **1** gave a positive reaction for a primary amino group (ninhydrin) (Figure 1).

The molecular formula of oceanalin B (**1**) was established as C_40_H_70_N_2_O_9_ on the basis of NMR and HRFABMS data (*m*/*z* 723.5286 [M + H]^+^, calcd. 723.5311 for C_40_H_71_N_2_O_9_). An intense peak in the ESIMS spectrum due to the doubly protonated molecular ion at *m*/*z* 362.7 [M + 2H]^2+^ (100%) was characteristic of α,ω-bifunctionalized sphingolipids [4,5]. Initial analysis of NMR data (Table 1, Supplementary Materials Figures S1–S5) showed signals of a hexose residue (*δ*_H_ 4.32, 3.51, 3.47, 3.78, 3.54, 3.72 and 3.74; *δ*_C_ 104.6, 73.3, 75.1, 71.1, 77.6 and 63.6), six aromatic carbons, two of which were protonated (*δ*_H_ 6.61 and 6.64; *δ*_C_ 124.2, 116.8, 147.3, 146.5, 114.5 and 124.8), a nitrogen-substituted CH_2_ (*δ*_H_ 3.50; *δ*_C_ 41.6), two N-substituted CH (*δ*_H_ 4.32 and 3.17; *δ*_C_ 57.3 and 52.7), two oxygenated CH (*δ*_H_ 3.67 and 3.94; *δ*_C_ 81.0 and 74.3), a disubstituted double bond (*δ*_H_ 5.39 and 5.58; *δ*_C_ 135.1 and 133.1) and a secondary methyl group (*δ*_H_ 1.27; *δ*_C_ 16.0) (Table 1). The remainder of the ^1^H NMR signals of **1** were attributed to a polymethylene chain (*δ*_H_ 1.27–1.29, brs). The ^1^H NMR data of **1** were similar to those of oceanalin A (**2**), except for the lack of OMe singlet (*δ*_H_ 3.20). One of the CH-O multiplets was shifted downfield from *δ*_H_ 3.50 to 3.94 [4]. Consequently, the structure of **1** was formulated as that of a homolog of oceanalin A with hydroxylation at C-18 that was subsequently confirmed by analysis of ^13^C NMR (*δ*_C_ 133.1, 135.1, 74.3), COSY and HMBC data (Figure 1). HMBC correlations allowed placement of this hydroxyl group at the allylic position of **1**. The galactopyranosyl residue in **1**, revealed by 2D NMR experiments, has the β-configuration at the anomeric carbon in concordance with the H-1′ coupling constant (*δ*_H_ 4.32, d, *J* = 7.2 Hz). The cross peak with C-3 (*δ*_C_ 81.0) in the HMBC spectrum established the attachment of the monosaccharide to this position.

**Figure 2:**
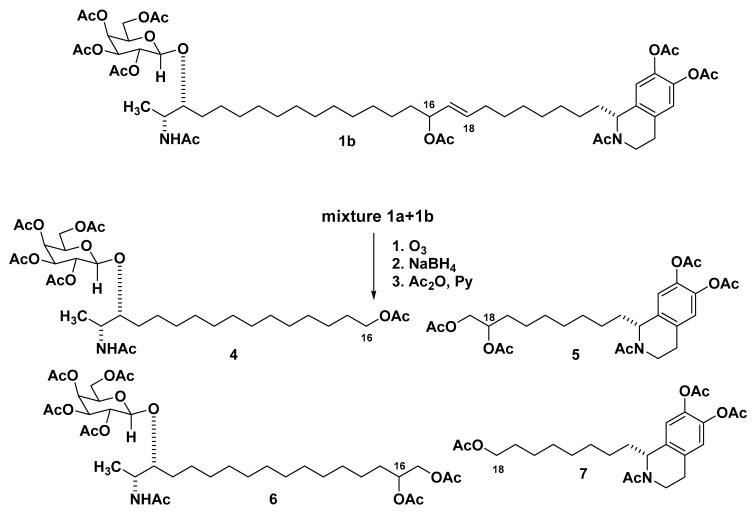
The structures of regioisomer oceanalin B peracetate **1b** and derivatives **4**–**7** obtained from oceanalin B by reductive ozonolysis and acetylation.

A successful solution to the problem of positioning the CH=CH-CH(OH) fragment in **1** was achieved as follows. Peracetylation of a crude mixture containing **1** and rhizochalin (**3**) peracetates followed by separation using HPLC (YMC-Pack ODS-A column, EtOH–H_2_O, 80:20) gave mainly oceanalin B peracetate **1a**. This peracetate was subjected to reductive ozonolysis (O_3_, then NaBH_4_) followed by acetylation (Ac_2_O, pyridine) [12]. Two products were isolated by HPLC as a mixture and identified as peracetates **4** and **5**, the same derivatives of oceanalin A [4] (Figure 2). NMR data confirmed that compound **4** retains the glycosylated terminus, while the derivative **5** contains a tetrahydroisoquinoline substituted polymethylene chain terminated by a 17,18-di-*O*-acetyl unit. The sodiated adduct ion peaks [M + Na]^+^ observed at *m*/*z* 710 and 556 in the MALDI-TOF MS spectra of compounds **4** and **5**, respectively, permitted us to determine the position of the allylic hydroxyl group as shown in the formula of **1**.

It seems that oceanalin B occurs naturally as a mixture of the two regioisomeric allylic alcohols, which appear to have identical NMR spectra due to the remote location of the functional group from the chain termini, so the mixture of peracetates **1a** + **1b**, obtained from oceanalin B, gave after ozonolysis-reduction peracetates **4** and **5** as major products along with the corresponding minor isomers **6** and **7** the same derivatives as oceanalin A [4] (Figure 2): the latter product presumably arising from **1b**. The observation of sodiated adduct ion peaks at *m*/*z* 782 [M + Na]^+^ and 484 [M + Na]^+^ in MALDI-TOF MS spectra of products **6** and **7**, respectively, confirmed their structures.

**Figure 3:**
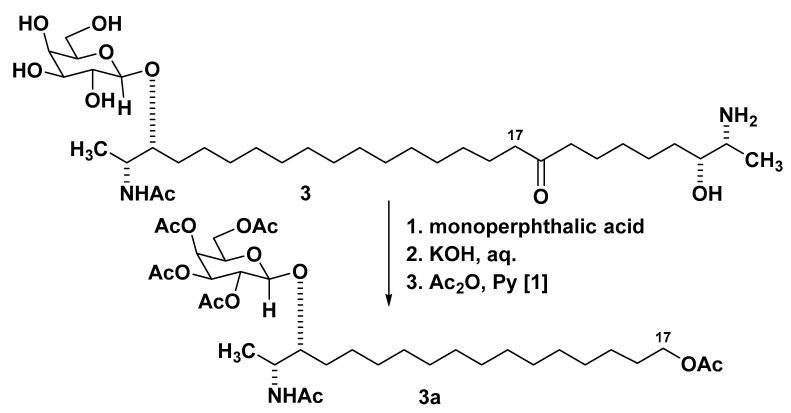
The structures of rhizochalin (**3**) and its derivative **3a**.

The absolute configuration of **1** was addressed as follows. Hydrolysis of **1** (2M HCl, 80 °C, 18 h) gave D-galactose. The ozonolysis product **4** obtained from **1a** was indistinguishable (NMR, [α]_D_^25^) from its known homolog **3a**, earlier obtained by another variant of oxidative degradation from rhizochalin (**3**) [1] (Figure 3). From the known absolute configuration of **3** [13], it is deduced that the absolute configuration of **4** is the same as that of **3a**. It may be concluded that oceanalin B (**1**) has the (2*R*,3*R*) configuration—the same as rhizochalin (**3**) and oceanalin A (**2**).

**Figure 4:**
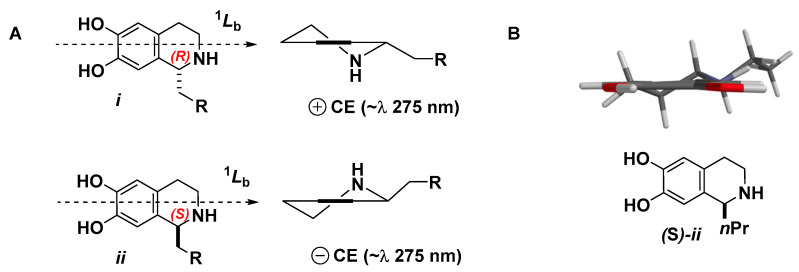
(**A**) Cotton effect (CE) in 1-substituted 6,7-dihydroxytetrahydroisoquinolines [14,15]. (**B**) MMFF minimized geometry of the lowest-energy conformer (Spartan ’18) of model tetrahydroisoquinoline ***ii*** conforms to Snatzke’s *C*_2v_ conformer of (*S*)-anhalonine ([14] and Figure 4A*ii*, R = Et).

Oceanalin B peracetate **1a** shows a positive benzenoid Cotton effect ([*θ*] + 300, λ_max_ 275 nm) that is associated with the ^1^*L*_b_ transition of the substituted dihydroxy-tetrahydroisoquinoline ring in the preferred C_2v_ conformation ***i*** (Figure 4) of saturated ring [14,15]. Therefore, the C-26 asymmetric center has the *R*-configuration.

It is interesting to note that oceanalin A (**2**), which appears to derive from **1** by acid-catalyzed methanolysis, displays no Cotton effect at this wavelength (~275 nm) and was assigned as a 1:1 epimeric mixture at C-26. Electron-rich 1-substituted 6,7-tetrahydroquinolines are well-known to undergo acid-catalyzed racemization by a retro-Pictet–Spengler reaction [4]; in the case of oceanalin A, the compound appears to have done so, most likely in the presence of acid in the HPLC solvent (0.1% TFA).

Oceanalin B (**1**) is the first α,ω-bipolar sphingolipid-like natural product that contains a labile allylic hydroxyl group. Biogenetically and spectrally, **1** is closely related to oceanalin A (**2**) and presumably has the same stereochemical lability. Taking into consideration that **1**, due to allylic rearrangement, is easily interconverted into its isomer, differing by positions of a double bond and hydroxy group, the assignment of absolute configuration of the hydroxy-bearing stereocenter is moot.

Oceanalin B (**1**) showed antifungal activity against *Candida glabrata.* Confirm that your intended meaning is retained Confirm that your intended meaning is retained with a minimum inhibitory concentration (MIC) of 25 µg/mL, which is comparable to the activity of oceanalin A (MIC = 30 µg/mL) [4] when tested under similar conditions. Due to its similarity in the structure of oceanapiside as a α,ω-bipolar compound with sphingolipid units on the ends of the molecule, oceanalin B may target the sphingolipid pathway of *C. glabrata* as demonstrated by the mechanism of action of oceanapiside [11].

In conclusion, we would like to emphasize that oceanalin B is an unusual and rare α,ω-bifunctionalized long-chain compound with isoquinoline moiety, isolated from sponges. Diverse metabolites with a chain length from C24 to C34 are characteristic of sponges belonging to the Demospongiae class. These include so-called demospongic fatty acids [16,17]. A group of rarer α,ω-bifunctionalized metabolites from sponges are not limited to sphingolipid-like derivatives such as rhizochalins and oceanalins; several other small series of bipolar natural compounds sponges are also known [18]. On the other hand, tetrahydroisoquinoline moieties are almost as rare as bipolar lipids among marine natural products in sponges, although they are widespread in terrestrial biologic sources. It is considered that some naturally occurring tetrahydroquinolines are derived from the condensation of tyramine, phenylethylamine or dopamine, with the corresponding aldehydes or α-ketocarboxylic acids (in analogy with a Pictet–Spengler reaction) followed by decarboxylation [19].

## 3. Materials and Methods

### 3.1. General Procedures

Optical rotations were measured using a Perkin-Elmer 343 polarimeter (Waltham, MA, USA). The circular dichroism (CD) spectra were recorded on a Jasco J-500A (Jasco, Kioto, Japan) spectropolarimeter in quartz cells of 1 cm path-length with the following parameters: λ range, 200–300 nm; bandwidth, 1 nm; scan speed, 0.3 nm·s^−1^. The ^1^H and ^13^C NMR experiments were performed with a Bruker DRX-500 spectrometer (Bruker, Bremen, Germany) at 500.13 and 125.8 MHz, respectively, with TMS as internal standard. ESIMS mass spectra were obtained on a Surveyor MSQ Thermo Finnigan mass spectrometer (Thermo, Walthem, MA, USA), coupled to an Agilent 1100 series HPLC (Agilent Technologies, Santa Clara, CA, USA), or by direct infusion in MeOH containing HCOOH (0.1%). FAB mass spectra were provided by the University of California, Riverside, mass spectrometry facility. MALDI-TOF mass spectra were recorded on a Bruker Biflex III laser desorption mass spectrometer (Bruker, Bremen, Germany) coupled with delayed extraction using N_2_ laser (337 nm) on α-cyano-4-hydroxycinnamic acid as matrix.

Low pressure column liquid chromatography was performed using Polychrom-1 (powder Teflon, Biolar, Latvia), Sephadex LH-20 (Sigma Chemical Co., Goleta, CA, USA) and silica gel L (40/100 μm, Chemapol, Praha, Czech Republic); silica gel plates 4.5 × 6.0 cm, (5–17 μm, Sorbfil, Krasnodar, Russia) were used for thin-layer chromatography. Preparative HPLC for isolation and separation of sphingolipids was carried out using an Agilent Series 1100 Instrument (Agilent Technologies, Santa Clara, CA, USA) equipped with differential refractometer RID-DE14901810 on YMC Pack-ODS-A column (10 × 250 mm, 5 μm, 1.3 mL/min) in 80:20:0.1% MeOH:H_2_O:TFA or in 80:20 EtOH:H_2_O.

### 3.2. Animal Material

The sponge *Oceanapia* sp. (phylum Porifera, class Demospongiae, subclass Heteroscleromorpha, order Haplosclerida, family Phloeodictyidae) was collected in November 1990 at a depth of 48 m by dredging near Scott Reef, 192 km NNW of Broome, Western Australia (16°33′6 S; 121°07′1 E) during a scientific cruise aboard RV “Akademik Oparin” and identified by Dr. V.B. Krasokhin (G.B. Elyakov Pacific Institute of Bioorganic Chemistry FEB RAS, Vladivostok, Russia). A voucher specimen is kept under registration number PIBOC#012-200, the marine invertebrate collection of Pacific Institute of G.B. Elyakov Pacific Institute of Bioorganic Chemistry FEB RAS (Vladivostok, Russia).

### 3.3. Extraction and Isolation

The fresh specimen of the sponge *Oceanapia* sp. was immediately lyophilized and kept at −20 °C until required. The lyophilized sponge (327 g) was extracted with EtOH. The EtOH extract was concentrated (34.9 g) and partitioned between 90% EtOH and hexane. The aqueous layer (70% EtOH) was further partitioned with n-BuOH and the n-BuOH layer was concentrated to afford a brown solid (18.0 g). A portion of the solid (10.0 g) was separated by hydrophobic flash chromatography on Polychrom-1 with stepwise gradient elution using aqueous EtOH (0–100% EtOH:H_2_O). The sphingolipid fraction (ninhydrin positive) was eluted with 40% aqueous EtOH. A reversed-phase separation of the fraction by HPLC (YMC Pack-ODS-A column, 10 × 250 mm, 5 μm, 1.3 mL/min, 80% MeOH/0.1% TFA) gave oceanalin B (**1**) and rhizochalin (**3**) after concentration of the corresponding fractions under a stream of N_2_ at room temperature.

### 3.4. Compound Characterization Data

Oceanalin B (**1**): 12.2 mg (0.03%). Pale yellow glass; [α]_D_^25^ –6.3 (c 0.35; MeOH), HR FAB MS *m*/*z* 723.5286 [M + H]^+^ (calcd for C_40_H_71_N_2_O_9_, 723.5311). UV (MeOH), λ_max_ 236 nm (ε 5000), 286 nm (ε 2200). ESIMS *m*/*z* 723 (30%) [M + H]^+^, 362 (100%) [M + 2H]^2+^. MALDI-TOF-MS *m*/*z* 745 [M + Na]^+^. ^1^H and ^13^C NMR data, see Table 1.

### 3.5. Acetylation of Oceanalin B *(**1**)*: Oceanalin B Peracetate

*Method A.* The sphingolipid fraction (33.0 mg), after filtration through Polychrom-1, was dissolved in pyridine (0.3 mL) and acetic anhydride (0.3 mL) and allowed to stand at 25 °C for 18 h. Removal of the volatile materials gave a residue (30.0 mg) of mixture **1a** and minor sphingolipids. Separation of the mixture by preparative HPLC (YMC Pack-ODS-A column, 10 × 250 mm, 5 μm, 1.3 mL/min, 80:20 EtOH–H_2_O) gave 6.3 mg of 2.6 mg of oceanalin B peracetate (**1a**) and rhizochalin peracetate. Oceanalin B peracetate (**1a**): MALDI-TOF MS *m*/*z* 1123 [M + Na]^+^, [α]_D_^25^ +13 (*c* 0.17; MeOH), CD: [*θ*] +300, λ_max_ 275 nm. ^1^H NMR data, see Table 2.

*Method B.* A sample of **1** (0.8 mg) was dissolved in pyridine (0.1 mL) and acetic anhydride (0.1 mL) and allowed to stand at 25 °C for 18 h. Removal of the volatile materials gave a residue (0.8 mg) of (**1a** + **1b**), MALDI-TOF MS *m*/*z* 1123 [M + Na]^+^.

### 3.6. Hydrolysis of Oceanalin B *(**1**)*

An amount of 3.3 mg of oceanalin B (**1**) in 1 mL of 2 M HCl in MeOH was heated at 80 °C for 24 h in a sealed vial; after that, the solution was cooled and concentrated under a stream of N_2_. The residue was subjected to microcolumn chromatography (15 × 50 mm, silica gel) with elution MeOH–CHCl_3_ (1:4) to obtain 1-*O*-methyl-d-galactopyranosides (0.8 mg), identified by direct comparison with standard samples (NMR, optical rotation).

### 3.7. Ozonolysis of ***1a*** Obtained by Method A

Ozone was bubbled through a solution of **1a** (2.5 mg) in MeOH, at a temperature of –20 °C to −30 °C, for 2 h. The solution was cooled and treated with an excess of NaBH_4_ (5 mg). The mixture was left at room temperature overnight and quenched with acetic acid to pH = 7. The mixture was evaporated and the obtained residue was treated with Ac_2_O–pyridine (1:1, 0.3 mL) at room temperature overnight. After removal of the volatiles, the residue was separated by chromatography (silica gel), using ethyl acetate as eluent, to afford a mixture of products **4** + **5** (1.0 mg). Separation of the mixture by preparative HPLC (YMC Pack-ODS-A column, 10 × 250 mm, 5 μm, 1.3 mL/min, 80:20 EtOH–H_2_O) afforded the pure compounds **4** (0.3 mg) and **5** (0.3 mg).

Compound **4**: amorphous solid; [α]_D_^25^ 0 (c 0.03; CHCl_3_); MALDI-TOF MS *m*/*z* 710 [M + Na]^+^. ^1^H NMR data, see Table S2 ref. [4].

Compound **5**: amorphous solid; [α]_D_^25^ 0 (c 0.03; CHCl_3_); MALDI-TOF MS *m*/*z* 556 [M + Na]^+^. ^1^H NMR data, see Table S2 ref. [4]. 

### 3.8. Ozonolysis of Mixture ***1a*** + ***1b*** Obtained by Method B

Ozone was bubbled through a solution of mixture **1a** + **1b** (2.5 mg) in MeOH, at a temperature of −20 °C to −30 °C, for 2 h. The solution was cooled and treated with an excess of NaBH_4_ (5 mg). The mixture was left at room temperature overnight and quenched with acetic acid (to pH = 7). The mixture was then evaporated and the residue treated with Ac_2_O–pyridine (1:1, 0.3 mL) at room temperature overnight. After removal of the volatiles, the residue was separated by chromatography (silica gel) using ethyl acetate as eluent to afford a mixture of **4**–**7** (1.0 mg). Separation of the mixture by preparative HPLC (YMC Pack-ODS-A column, 10 × 250 mm, 5 μm, 1.3 mL/min, 80:20 EtOH–H_2_O) afforded the pure compounds **4** (0.3 mg), **5** (0.3 mg), **6** (0.1 mg) and **7** (0.1 mg).

Compound **6**: amorphous solid; [α]_D_^25^ 0 (c 0.01; CHCl_3_); MALDI-TOF MS *m*/*z* 782 [M + Na]^+^. ^1^H NMR data, see Table S2 ref. [4]. 

Compound **7**: amorphous solid; [α]_D_^25^ 0 (c 0.01; CHCl_3_); MALDI-TOF MS *m*/*z* 484 [M + Na]^+^. ^1^H NMR data, see Table S2 ref. [4].

### 3.9. Antifungal Activity

The fungal isolate used in this study was a *Candida glabrata* clinical isolate (University of California Davis Medical Center, UCDMC). The fungi were grown and maintained in Sabouraud dextrose agar and incubated at 30 °C for 24 h. The in vitro susceptibility of oceanalin B was determined by the broth microdilution method [20]. Briefly, 2-fold serial dilutions of oceanalin B were prepared in 96-well microtiter plates from stock solutions in an RPMI-1640 broth medium (Sigma, St. Louis, MO, USA) buffered to a final pH of 7.0 with 0.165 M morpholinepropanesulfonic acid (MOPS; Sigma, St. Louis, MO, USA) to a final volume of 100 μL. A stock solution of oceanalin B was prepared in dimethyl sulfoxide and amphotericin B (AMB) (Sigma, St. Louis, MO, USA) was prepared as a positive control. *C. glabrata* cells (5 × 10^6^/mL) in 100 μL suspension was added to the wells. The final concentrations tested were from 0.062 to 64 μg/mL and from 0.0078 to 8 μg/mL for amphotericin B. The experiment was performed in triplicates in each run of the experiments. Cell growth was determined by the OD at 600 nm using a Spectramax Plus 384 microplate reader (Molecular Devices, San Jose, CA, USA). The MIC end point was defined as the lowest concentration with complete (>90%) growth inhibition.

## Figures and Tables

**Table 1:** ^1^H (500.13 MHz) and ^13^C (125.8 MHz) NMR data for oceanalin B (**1**) (CD_3_OD; *δ* in ppm, *J* values in Hz).

Atom No.	*δ*_C_	*δ*_H_	COSY	HMBC
1	16.0	1.27 (d, 6.7)	H-2	C-2, C-3
2	52.7	3.17 (m)	H-1, H-3	
3	81.0	3.67 (ddd, 3.2, 7.2, 9.7)	H-2, H-4a	
4a	33.3	1.52 (m)	H-3	
4b		1.68 (m)		
5–13	30.8–31.6	1.27–1.29 (brs)		
14	31.2	1.37 (m)		
15	33.9	2.02 (m, 2H)	H-14	C-14, C-15, C-17
16	133.1	5.58 (dt, 7.0, 15.4)	H-17, H-15	C-15, C-18
17	135.1	5.39 (dd, 15.4, 7.0)	H-16, H-18	C-15, C-18
18	74.3	3.94 (q, 7.0)	H-17, H-19a,b	C-16
19a	39.1	1.42 (m)	H-18	
19b		1.50 (m)	H-18	
20–23	30.8–31.6	1.27–1.29 (brs)		
24a	27.2	1.36 (m)	H-25a	
24b		1.49 (m)	H-25a,b	
25a	35.7	2.02 (m)	H-24a,b, H-26	
25b		1.87 (m)	H-24b	
26	57.3	4.32 (dd, 4.6, 8.2)	H-25a,b	C-35
28a	41.6	3.50 (m)		
28b		3.50 (m)	H-29a,b	C-30
29a	26.3	2.89 (dt, 17.0, 6.0)	H-28b, H-29b	C-28, C-31, C-35
29b		2.97 (ddd, 6.5, 8.3, 17.0)	H-28b, H-29a	C-31, C-35
30	124.2	-		
31	116.8	6.61 (s)		C-29, C-33, C-35
32	147.3	-		
33	146.5	-		
34	114.5	6.64 (s)		C-26, C-32, C-30
35	124.8	-		
1′	104.6	4.32 (d, 7.2)	H-2′	C-3
2′	73.3	3.51 (dd, 7.2, 9.8)	H-3′, H-1′	
3′	75.1	3.47 (dd, 3.4, 9.8)		
4′	71.1	3.78 (d, 3.4)		
5′	77.6	3.54 (dd, 4.6, 6.5)		
6′	63.6	3.72 (m); 3.74 (m)		

**Table 2:** Selected ^1^H (500.13 MHz) NMR data for oceanalin B peracetate (**1a**) (CDCl_3_; TMS; *δ* in ppm, *J* values in Hz).

Atom No.	*δ*_H_	Atom No.	*δ*_H_
1	1.165 (d, 6.8)	31	6.93 (s)
2	4.09 (m)	32-OAc	2.28 (s)
2-NHAc	5.82 (d, 8.3)	33-OAc	2.27 (s); 2.29 (s)
3	3.49 (td, 2.7, 6.5)	34	6.94 (s)
6–13	1.25 (brs)	1′	4.48 (d, 8.0)
16	5.67 (m)	2′	5.16 (dd, 8.0, 10,6)
17	5.37 (m)	3′	5.04 (dd, 3.3, 10.6)
18	5.17 (m)	4′	5.39 (dd, 0.8, 3.3)
18-OAc	2.02 (s)	5′	3.91 (td, 0.8, 6.6)
20–24	1.25 (brs)	6′	4.10 (dd, 6.6, 11.3)
26	5.58 (dd, 5.5, 9.7)		4.19 (dd, 6.6, 11.3)
27-NAc	2.15 (s); 2.16 (s)	4xOAc	1.96 (s)
28a	3.78 (ddd, 4.0, 5.4, 13.6)		1.99 (s)
28b	3.52 (m)		2.04 (s)
29a	2.80 (m)		2.05 (s)
29b	2.90 (m)		

## Data Availability

Data is contained within the article or supplementary material.

## Supplementary Materials

The following are available online at https://www.mdpi.com/article/10.3390/md19110635/s1, Figure S1: ^1^H NMR spectrum of oceanalin B (**1**) in CD_3_OD. Figure S2: ^13^C NMR spectrum of oceanalin B (**1**) in CD_3_OD. Figure S3: 1H-1H-COSY spectrum of oceanalin B (**1**) in CD_3_OD. Figure S4: HSQC spectrum of oceanalin B (**1**) in CD_3_OD. Figure S5: HMBC spectrum of oceanalin B (**1**) in CD_3_OD.
