# Supplementary material for: Oceanalin B, a Hybrid α,ω-Bifunctionalized Sphingoid Tetrahydroisoquinoline β-Glycoside from the Marine Sponge Oceanapia sp
Source: Mar Drugs. 2021 Nov 12;19(11):635. doi: 10.3390/md19110635 (PMC8618332; doi:10.3390/md19110635)

Supporting Information

Oceanalin B, a Hybrid *α*,ω-Bifunctionalized Sphingoid Tetrahydroisoquinoline β-Glycoside from the Marine Sponge *Oceanapia* sp.

Tatyana N. Makarieva[1*], Natalia V. Ivanchina[1], Pavel S. Dmitrenok[1], Alla G. Guzii[1], Valentin A. Stonik[1*], Doralyn S. Dalisay[2,3] and Tadeusz F. Molinski[2]

[1] G.B. Elyakov Pacific Institute of Bioorganic Chemistry, Far Eastern Branch of the Russian Academy of Sciences, Pr. 100-let Vladivostoku 159, 690022, Vladivostok, Russian Federation; ivanchina@piboc.dvo.ru (N.V.I.); paveldmt@piboc.dvo.ru (P.S.D.); gagry@rambler.ru (A.G.G.); stonik@piboc.dvo.ru (V.A.S.)

[2] Department of Chemistry and Biochemistry/SSPPS, University of California, San Diego, La Jolla, CA 92093-0358, U.S.A.; tmolinski@ucsd.edu (T.F.M.)

[3] Center for Chemical Biology and Biotechnology (C2B2) and Department of Biology, College of Liberal Arts, Sciences and Education, University of San Agustin, Iloilo City 5000, Philippines; ddalisay@usa.edu.ph (D.S.D.)

* Correspondence: makarieva@piboc.dvo.ru (T.N.M.); stonik@piboc.dvo.ru (V.A.S.); Tel.: 8(423)231-11-68; Fax: 8(423)231-40-50.

Figure S1. ¹H NMR spectrum of oceanalin B (**1**) in CD_3OD.

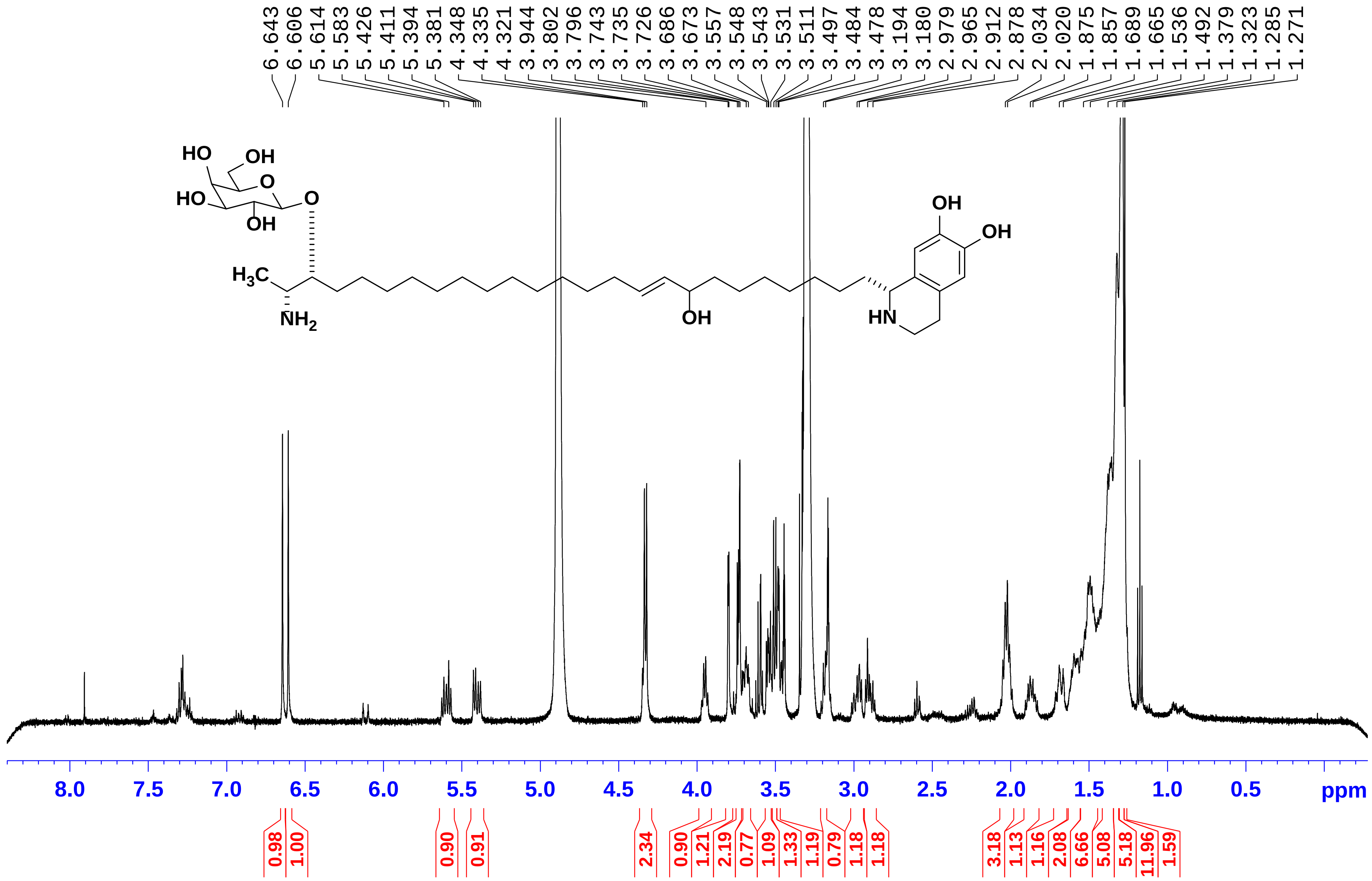

Figure S2. ^{13}C NMR spectrum of oceanalin B (**1**) in CD_3OD.

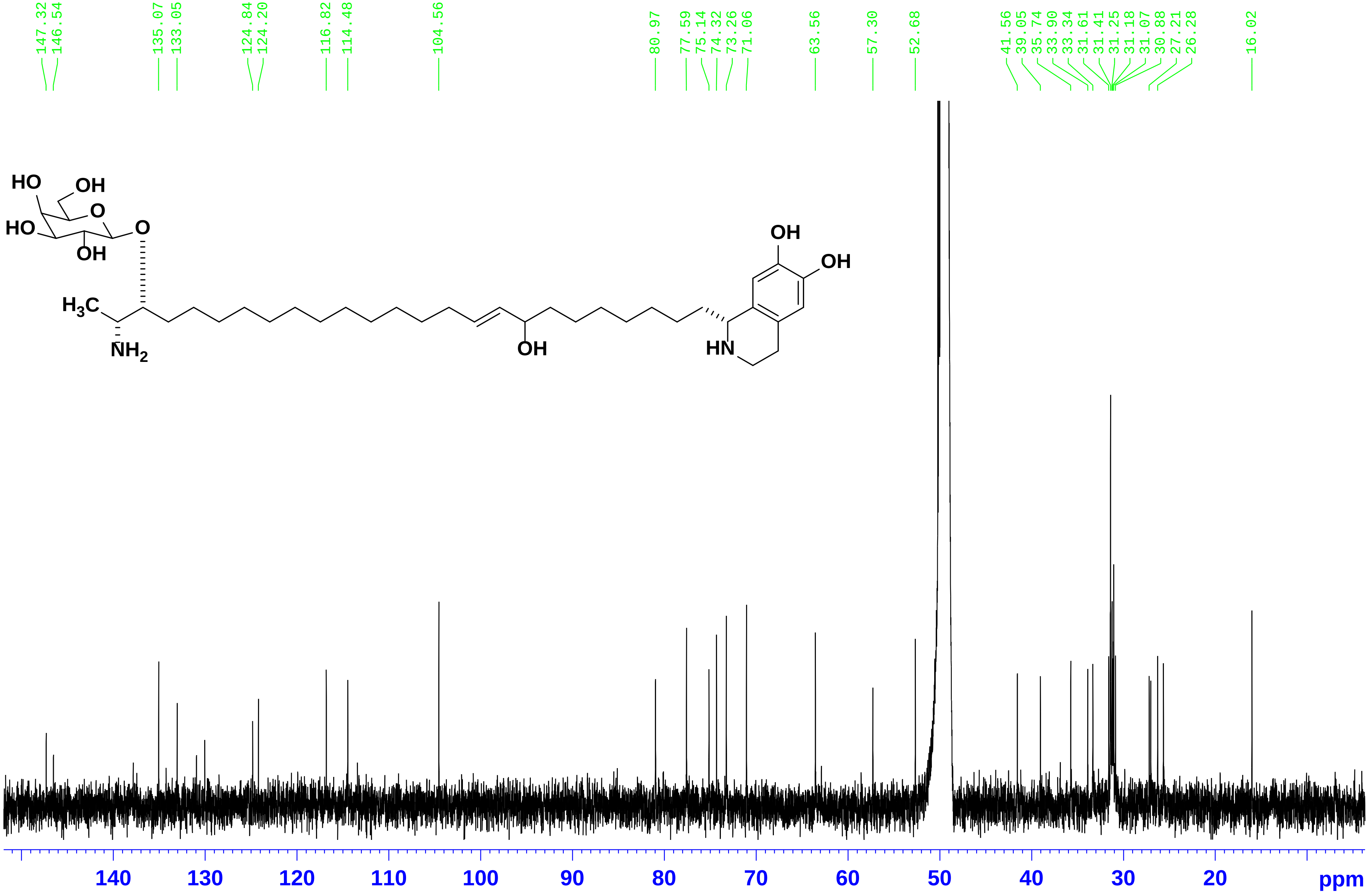

Figure S3. ^{1}H-^{1}H-COSY spectrum of oceanalin B (**1**) in CD_3OD.

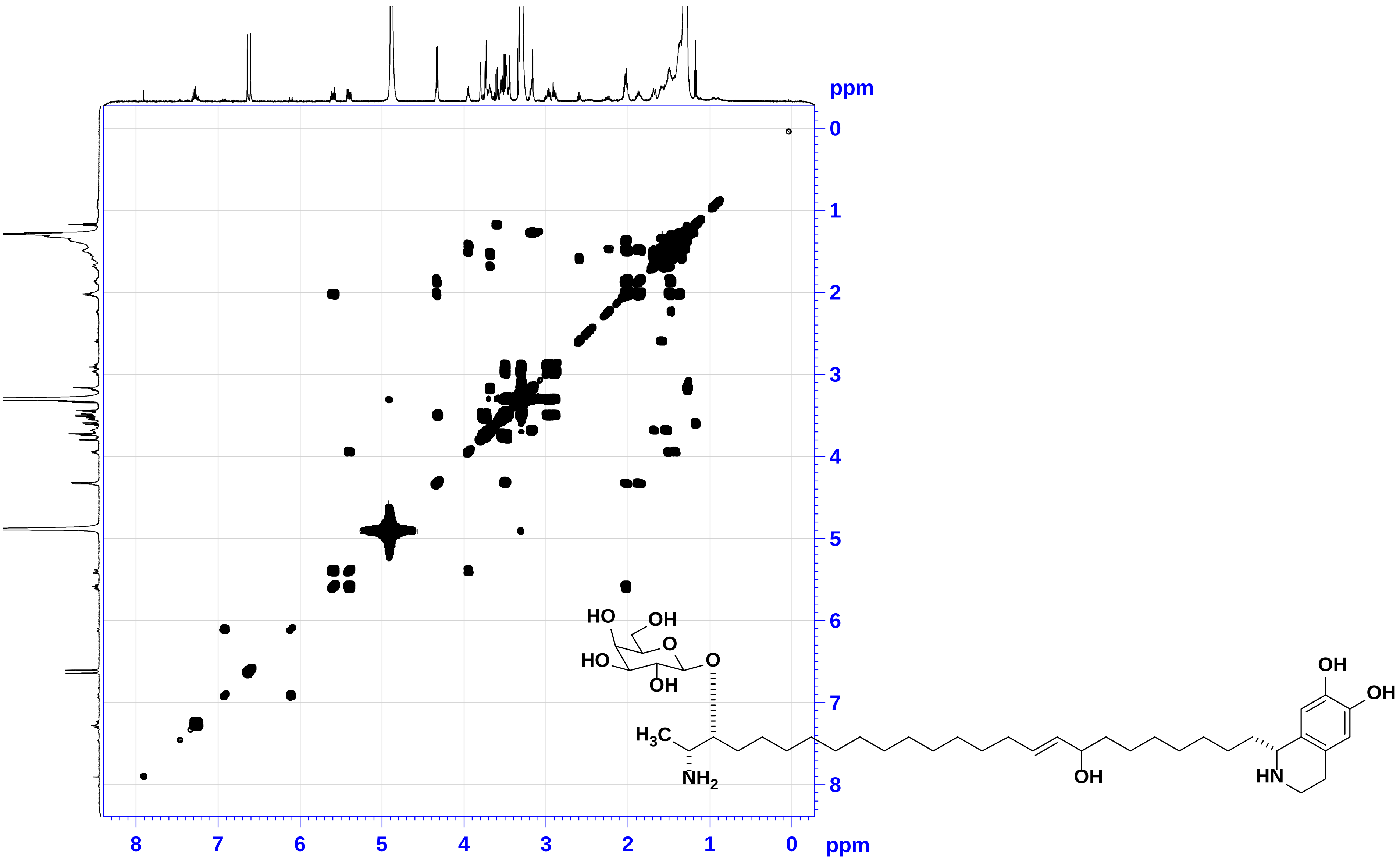

Figure S4. HSQC spectrum of oceanalin B (**1**) in CD_3OD.

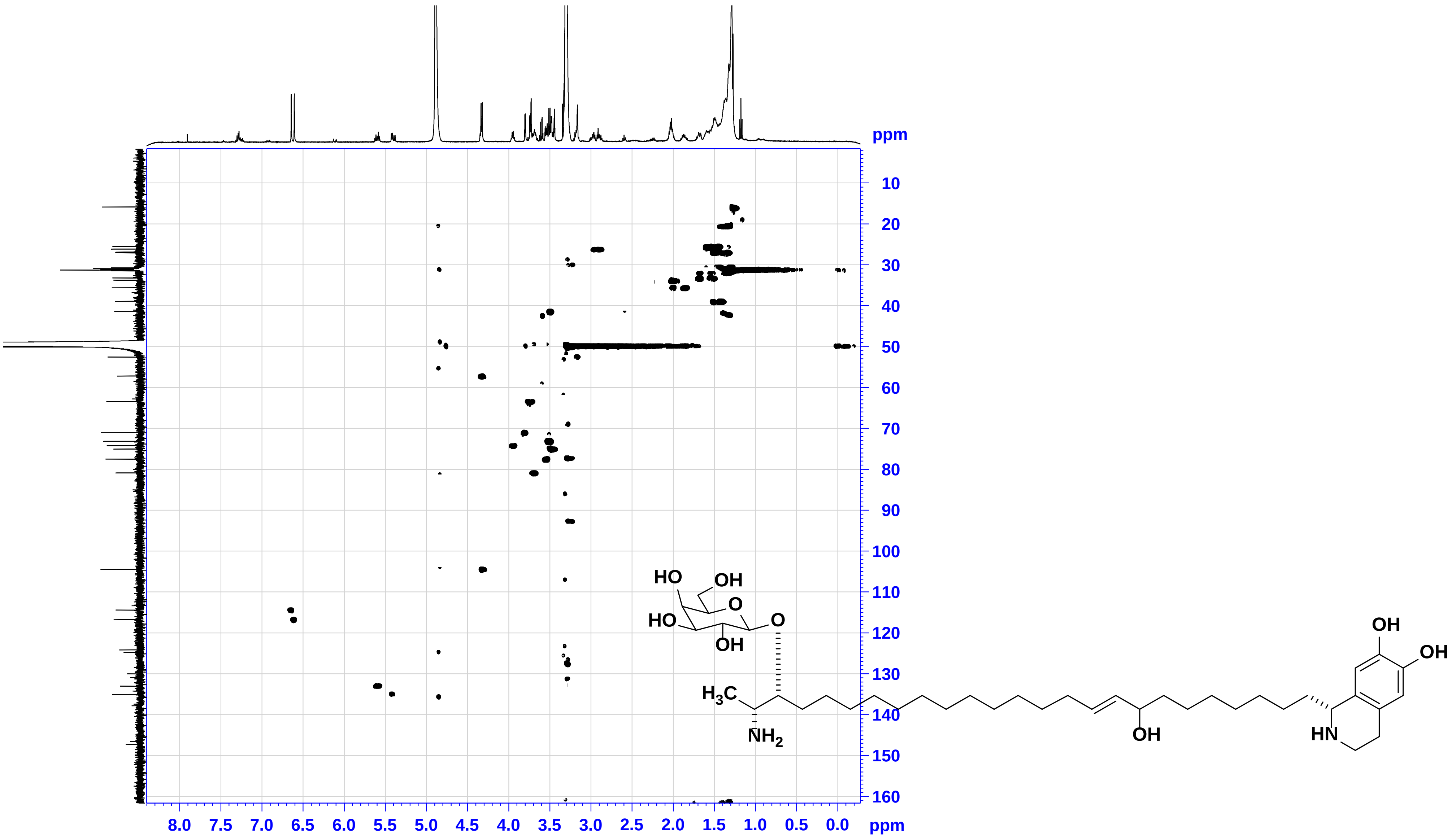

Figure S5. HMBC spectrum of oceanalin B (**1**) in CD_3OD.

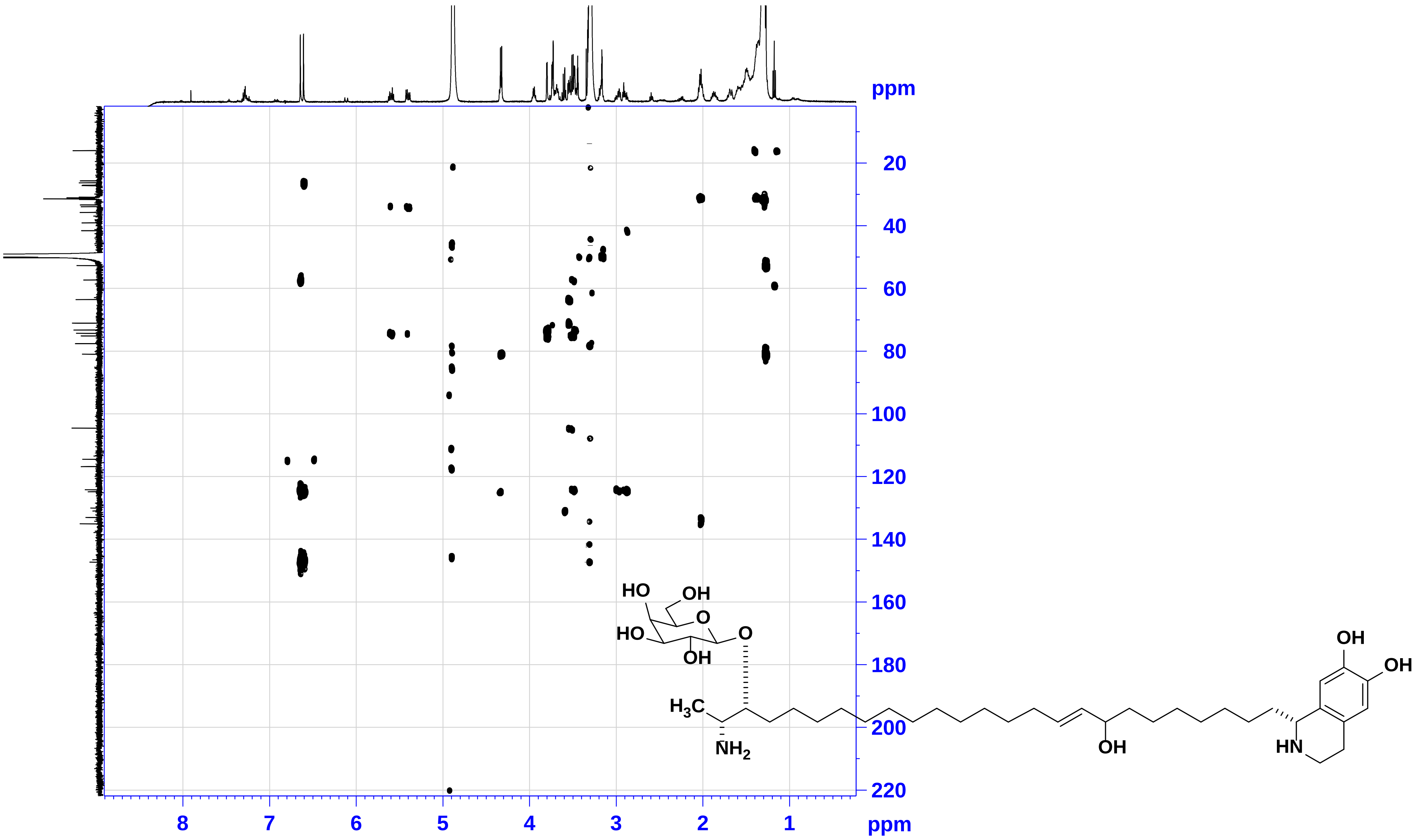

Supplement: Supplementary file 1 [file marinedrugs-19-00635-s001.zip › marinedrugs-1452433-supplementary.pdf]
